# RNA, Genome Output and Input

**DOI:** 10.3389/fgene.2020.589413

**Published:** 2020-10-27

**Authors:** Jörg Morf, Srinjan Basu, Paulo P. Amaral

**Affiliations:** ^1^Jeffrey Cheah Biomedical Centre, Wellcome – Medical Research Council (MRC) Cambridge Stem Cell Institute, University of Cambridge, Cambridge, United Kingdom; ^2^Jeffrey Cheah Biomedical Centre, The Milner Therapeutics Institute, University of Cambridge, Cambridge, United Kingdom

**Keywords:** RNA, lncRNA, gene regulation, chromosome conformation, transcriptional bursting

## Abstract

RNA, the transcriptional output of genomes, not only templates protein synthesis or directly engages in catalytic functions, but can feed back to the genome and serve as regulatory input for gene expression. Transcripts affecting the RNA abundance of other genes act by mechanisms similar to and in concert with protein factors that control transcription. Through recruitment or blocking of activating and silencing complexes to specific genomic loci, RNA and protein factors can favor transcription or lower the local gene expression potential. Most regulatory proteins enter nuclei from all directions to start the search for increased affinity to specific DNA sequences or to other proteins nearby genuine gene targets. In contrast, RNAs emerge from spatial point sources within nuclei, their encoding genes. A transcriptional burst can result in the local appearance of multiple nascent RNA copies at once, in turn increasing local nucleic acid density and RNA motif abundance before diffusion into the nuclear neighborhood. The confined initial localization of regulatory RNAs causing accumulation of protein co-factors raises the intriguing possibility that target specificity of non-coding, and probably coding, RNAs is achieved through gene/RNA positioning and spatial proximity to regulated genomic regions. Here we review examples of positional cis conservation of regulatory RNAs with respect to target genes, spatial proximity of enhancer RNAs to promoters through DNA looping and RNA-mediated formation of membrane-less structures to control chromatin structure and expression. We speculate that linear and spatial proximity between regulatory RNA-encoding genes and gene targets could possibly ease the evolutionary pressure on maintaining regulatory RNA sequence conservation.

## Introduction

Many mechanisms for RNAs to regulate gene expression in the cell nucleus involve recruitment of regulatory protein factors, including chromatin modifiers and polymerase recruiters that affect the transcriptional output of genes (Ulitsky and Bartel, 2013). Non-coding RNAs above the length range of small RNAs exemplified by miRNAs and clearly distinguishable from transcription units of protein-coding loci, i.e., intergenic, have been studied extensively in the past. The definition of and focus on long non-coding RNAs (lncRNAs) facilitates the functional characterization of how RNA molecules affect the expression of genes whilst avoiding ambiguities arising from the bifunctionality of coding RNAs, i.e., an RNA with regulatory potential simultaneously encoding a protein with a certain function. However, it is unlikely that most nuclear complexes and machineries with affinity toward RNA distinguish transcripts primarily based on their coding potential, and roles in regulatory relationships between coding and non-coding transcripts could therefore be assumed as interchangeable (Li and Liu, 2019).

Most regulatory proteins, such as transcription factors, enter nuclei after their synthesis through pores to eventually interact specifically, or broadly, with chromatin regions. RNAs, on the other hand, emerge from their encoding gene at a defined genomic and spatial position. Therefore, the position of origin for a regulatory RNA and the spatial genome neighborhood are arguably critical in defining target gene specificity for such transcripts. Particularly RNAs with shorter half-lives might exert roles in gene expression regulation restricted to nuclear regions in immediate vicinity to their gene locus encompassing neighboring genes in cis or loci brought into close proximity in the spatial genome structure through chromatin looping or DNA contacts in trans.

Once a polymerase engages in processive transcription, the synthesized, nascent transcript appears from the encoding gene locus. The RNA molecule grows in length with continuing transcription along the gene but stays tethered to chromatin by the polymerase until 3’ RNA cleavage followed by polyadenylation releases the RNA molecule (Cramer, 2019). At a polymerase elongation rate usually between 1 and 4 kilobases per minute and a median gene length of around 24 kilobases in human cells, at least the 5’ region of a nascent transcript is extruding chromatin while still tethered to it for a duration in the order of 10 min (Milo et al., 2010). Immediately after initiation of RNA synthesis, proteins with RNA-binding domains can interact co-transcriptionally with 5’ ends of nascent transcripts. Interestingly, sequence conservation of non-coding RNAs, which is overall low compared to mRNAs, increases toward the 5’ ends of the molecules, which raises the possibility that the longer half-life in chromatin association of 5’ RNA regions compared to 3’ ends has been co-opted to more efficiently recruit regulatory protein factors to chromatin through interactions with nascent, tethered RNA (Hezroni et al., 2015).

Nascent RNAs emerge as groups of multiple molecules in a short time window, so-called transcriptional bursts, which results in the amplification of available chromatin-tethered RNA binding sites and of protein recruitment to a given locus in that moment. Bursting, or discontinuous transcription, of active genes describes the temporal gating of transcription initiation into time windows of a few minutes and the interspersion of such “on” states with longer periods of promoter inactivity, or so-called “off” states (Rodriguez and Larson, 2020). First insights into discontinuous transcription have been gained by electron microscopy of chromosome spreading preparations (McKnight and Miller, 1979). More recently, bursting parameters such as frequency and burst size, the number of transcription initiations during a burst, have been measured by single-molecule RNA-FISH, short-lived protein reporters, MS2-RNA tagging and single-cell RNA-seq (McKnight and Miller, 1979; Raj et al., 2006; Suter et al., 2011; Tantale et al., 2016; Larsson et al., 2019). Typically, bursts measured in mammalian cells have frequencies in the order of one burst every 30 min up to several hours and last for a couple of minutes. RNA polymerases start transcribing in groups with inter-polymerase distances of a few hundred bases during on states, which gives rise to up to hundreds of nascent transcripts emerging from and tethered to chromatin during the time required for polymerases to reach the 3’ end of a gene (Dar et al., 2012; Tantale et al., 2016; Nicolas et al., 2017).

Intriguingly, a recent preprint applying RNA-FISH combined with expansion microscopy revealed that transcripts after completion of synthesis and chromatin dissociation remain locally restricted within sub-micron distances from gene loci for some time (Coté et al., 2020). The absence of gradients of decreasing RNA concentration from the encoding gene contrasts the notion of immediate free diffusion or transport away from genes after transcription termination and 3’ RNA end processing. Such a delay in transcript re-localization after synthesis would further increase the chromatin residence time of transcripts.

Once regulatory transcripts escape localization to the vicinity of their encoding gene locus, the target gene search is expected to rely primarily on differential affinities to for example different DNA sequences, chromatin modifications and other chromatin-associated factors, comparable to regulatory proteins entering the nucleus through pores.

**FIGURE 1:**
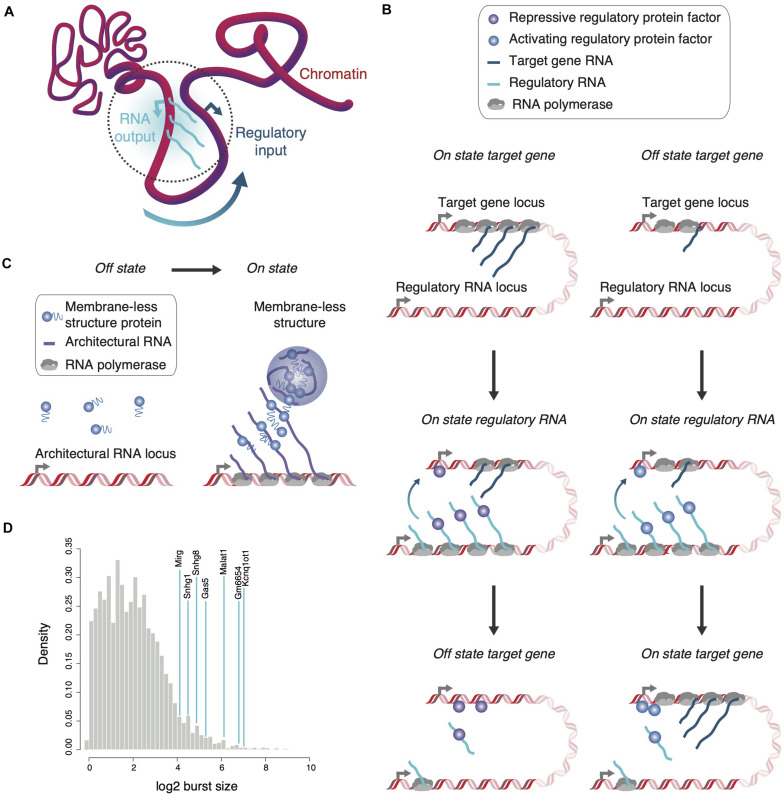
Localization and enrichment of regulatory or structural proteins by RNA. **(A)** The transcriptional output of a regulatory RNA-encoding gene can serve as regulatory input for genes in spatial proximity. Various parameters including transcription kinetics and chromatin association might define the spatial reach of the regulatory RNA (dotted gray circle). **(B)** Regulatory RNAs can affect expression of proximal genes either negatively (left) or positively (right). During a burst, the appearance of multiple, chromatin-associated regulatory RNAs attracts and localizes protein factors in proximity to the target gene to increase or decrease its transcriptional output. **(C)** Architectural RNAs with large burst sizes and multiple affinity sites for structural proteins, which form multivalent interactions when concentrated in close proximity, are envisaged to seed membrane-less structures co-transcriptionally. **(D)** The large burst sizes of known non-coding RNA genes (blue lines) are highlighted in comparison to burst sizes of all protein-coding genes (gray distribution, data from Larsson et al., 2019). Of note, despite overall lower burst frequencies for ncRNAs than protein-coding RNAs, distributions in burst size are similar (Kouno et al., 2019; Larsson et al., 2019).

In summary, transcriptional bursts locally increase RNA concentration throughout the time of synthesis when RNA is tethered to chromatin and likely longer in an untethered state in the immediate vicinity of encoding genome loci. As a consequence, a high density of locally confined single-, double-stranded and structural RNA motifs presents itself at transcription units to concentrate and position nucleic acid-binding proteins with gene regulatory functions within the three-dimensional genome structure (Figures 1A–C). Indeed, many transcription factors have RNA-binding capacity and, vice versa, nuclear RNA-binding proteins are frequently found localized to chromatin (Cassiday and Maher, 2002; Hudson and Ortlund, 2014; Xiao et al., 2019).

The information of a single allelic genome motif can therefore be locally amplified in the orders of 10–100-fold when converted into RNA during a single transcriptional burst. However, whether the regulatory potential of nascent RNA is realized might depend on factors that, besides transcription and degradation kinetics, include the sequence and structural features of the RNA, RNA modifications and, importantly, linear or spatial proximity to potential target loci. In the following paragraphs we revisit a few illustrative examples of regulatory RNAs and their effects on the transcriptional output of genes encoded in close distance on the same chromosome or in proximity either through chromatin looping or gene positioning to nuclear bodies.

## Positional Conservation of Cis-Regulatory RNAs and Target Genes

A case in point is illustrated by lncRNAs whose genomic position relative to neighboring genes is conserved (synteny) (Ulitsky, 2016). The classic lncRNA *Xist*, which forms RNA clouds covering exclusively the inactive X chromosome was the first regulatory lncRNA found to display genomic positional conservation across species (Brockdorff et al., 1991; Brown et al., 1991, 1992). Thereafter, thousands of additional non-coding RNAs, whose genomic position, promoters and tissue expression profiles are conserved between human and mouse, have been identified and named positionally-conserved RNAs (pcRNAs) (Amaral et al., 2018). Most pcRNAs locate to chromatin loop anchor points and borders of topologically associating domains (TADs), occupied by the CTCF chromatin organizer and of increased gene density in cis and 3D for lncRNAs to act upon (Kim et al., 2007). Their expression is correlated to the abundance of transcripts from neighboring genes, which are predominantly developmental genes. Experimental reduction of the RNA levels of several of these pcRNAs in different human and cancer cell lines leads to downregulation of the syntenic protein-coding gene, indicating positive regulatory roles in cis for pcRNAs. Indeed, co-expression and cis-regulation of associated genes by neighboring lncRNAs is a common mode of regulation (Guil and Esteller, 2012; Gil and Ulitsky, 2020).

The example of pcRNA *Evx1-as* illustrates a possible sequence of steps that leads to the upregulation of *Evx1* target transcription in cis. *Evx1-as* pcRNA and *Evx1* coding transcripts are co-expressed in the primitive streak of mouse embryos (Bell et al., 2016). The lncRNA first recruits MLL chromatin modifiers, which deposit locally H3K4me3 histone marks upon differentiation of mouse embryonic stem cells toward mesoderm (Dinger et al., 2008). Increased H3K4me3 levels and subsequent recruitment of transcriptional activators, such as the Mediator complex, is followed by the induction of *Evx1* transcription (Luo et al., 2016). The importance of the proximity between the lncRNA and the target gene is highlighted by gain-of-function experiments. Consistent with a genome position-dependent role, ectopic over-expression and mis-localization of *Evx1-as* does not affect *Evx1* levels (Luo et al., 2016). Reduction in *Evx1-as* RNA levels phenocopies loss-of-function of *Evx1* (Bell et al., 2016; Luo et al., 2016). Similar modes of action have been identified for *HOTTIP, HoxBlinc*, and *HOTAIRM1* regulating multiple neighboring *HOX* genes in expression domains during cell differentiation (Zhang et al., 2009; Wang et al., 2011; Deng et al., 2016; Wang and Dostie, 2017) and for lncRNAs, such as *UMLILO*, priming the robust expression of proximal immune genes (Fanucchi et al., 2019).

Interestingly, in some cases the lncRNA and the neighboring gene act in concert in feedback loops. LncRNA *Deanr1*, encoded adjacent to *Foxa2*, recruits SMAD2/3 transcription factors to the *Foxa2* promoter to activate coding gene transcription (Luo et al., 2016). Reciprocally, reduction of *Foxa2* coding RNA results in decreased expression levels of its neighboring lncRNA. It is unclear to date whether *Foxa2* RNA mediates the effect on lncRNA expression or whether FOXA2 protein and its chromatin binding sites at its own promoter and that of the lncRNA reinforces the expression of both (Amaral et al., 2018).

As exemplified by *Xist*, cis-acting RNAs may also function as negative regulators of neighboring gene expression. Repression in *cis* commonly underlies regulation of imprinted loci associated with positionally conserved lncRNAs—as for the example of *Kcnq1ot1* and other lncRNAs, such as *Airn* and *H19* (Barlow and Bartolomei, 2014; Schertzer et al., 2019). The large transcriptional burst size of *Kcnq1ot1* and a transcript length of almost 100 kb, which requires an estimated half an hour to complete transcription, likely contribute to the generation of micrometer-large *Kcnq1ot1* RNA clouds (Figure 1C). Such tethered RNA sponges efficiently recruit polycomb repressive complexes to silence neighboring genes allele-specifically over megabase distances (Murakami et al., 2007; Mohammad et al., 2008; Redrup et al., 2009; Larsson et al., 2019; Schertzer et al., 2019). RNA has been found as a key determinant for the association of Polycomb repressive complex 2 (PRC2) with chromatin. PRC2 is recruited to chromatin through interactions with nascent RNAs as well as evicted from it upon interaction with G-tracts in RNAs (Beltran et al., 2019; Long et al., 2020).

*Xist*, on the other hand, provides an example of a lncRNA that covers a larger chromatin territory, a whole X chromosome. Such a reach is unlikely to be achieved only through co-transcriptional tethering of the RNA to the *Xist* locus by RNA polymerases, and *Xist* interactions with hnRNP U/SAF-A and CIZ1 are believed to contribute to X chromosome association of the RNA beyond the immediate vicinity of the *Xist* transcription site (Hasegawa et al., 2010; Ridings-Figueroa et al., 2017; Sunwoo et al., 2017). Other post-transcriptional or polymerase-independent mechanisms to retain regulatory transcripts on chromatin include the hybridization of RNA to a complementary region in one strand of melted DNA to form R loops, Hoogsteen base-pairing resulting in RNA:DNA triplexes, or tethering of RNA to chromatin by U1 snRNP in a splicing-independent manner (Chédin, 2016; Li et al., 2016; Yin et al., 2020). *Xist*-directed dosage compensation in female mammals depends on the recruitment and eviction of regulatory protein complexes through repeated RNA motifs and structures in *Xist*, which show increasing evolutionary conservation toward the 5’ end of the transcript (Brown et al., 1992; Colognori et al., 2020; Strehle and Guttman, 2020). Inactivation of the X chromosome initiates at the site of *Xist* transcription and then extends with *Xist* spreading to proximal chromosomal regions and subsequently to more distal sites according to a “first come first served” principle in three-dimensional space (Engreitz et al., 2013). After initial coating of the chromosome by *Xist*, RNA-binding proteins, while interacting simultaneously with a repeat region in *Xist*, are believed to form condensates to sustain anchoring of *Xist* to the inactivated X territory and X chromosome silencing (Pandya-Jones et al., 2020).

## Spatial Proximity Between Enhancer RNAs and Promoters

Enhancers are regulatory genomic elements, which modulate the expression of genes in linear and spatial proximity. Some key features of potent enhancers resemble those of active genes: open chromatin, certain shared chromatin modifications and promoter elements, RNA polymerase binding and the synthesis of RNA (Andersson and Sandelin, 2020). Enhancer-derived RNAs (eRNAs) are frequently short-lived, which coincides with their local restriction to corresponding, transcript-encoding enhancer regions. eRNAs have been implicated in the regulation of target genes by enhancers and different studies have shown that eRNA transcription precedes target mRNA transcription, a prerequisite to initiate first steps of target gene transcription (Arner et al., 2015; Kim et al., 2015; de Lara et al., 2019). It is believed that enhancer chromatin regions and associated eRNAs are placed into proximity of target promoters for gene activation, although different studies on the correlation of the time of DNA looping and gene activation reached different conclusions (Lai et al., 2013; Alexander et al., 2019; Benabdallah et al., 2019; Schoenfelder and Fraser, 2019).

Like other transcripts, eRNAs are synthesized discontinuously, and evidence suggests that eRNAs in turn can modulate transcriptional bursting of target genes. Single-cell sequencing analysis of enhancer expression revealed that estimates of burst size in eRNA transcription matches those of genes (Kouno et al., 2019; Larsson et al., 2019). However, the frequency of bursts is lower for enhancers than for genes, contributing to an overall lower cell population-averaged RNA signal for enhancers than for genes. To gain insights into the regulatory relationship between eRNAs and target RNAs, Rahman et al. determined abundance and co-localization of both upon estrogen signaling and using fluorescence *in situ* hybridization (FISH). Estrogen treatment increases the number of cells, i.e., the burst frequency, that express eRNAs and the corresponding, estrogen-responsive target genes *Foxc1* and *P2ry2*. Co-localization of eRNA and nascent target gene RNA spots increases from less than 5% in unstimulated cells around 5-fold to 25% after estrogen treatment (Rahman et al., 2017). Importantly, the size of RNA-FISH spots of *Foxc1*, a proxy for burst size, was found increased in cells with co-localization of *Foxc1* and eRNA. However, the details of the underlying mechanism remain to be fully resolved.

The transcription factor Yin Yang 1 (YY1) and its interactions with eRNAs serves as one mechanistic example of how regulatory RNAs contribute to target gene regulation. YY1 binds to active enhancers and promoters and forms dimers to stabilize DNA looping (Weintraub et al., 2017). Different regions of the YY1 protein bind to DNA and RNA, respectively, at those regulatory elements. Upon experimentally decreasing the abundance of enhancer RNAs YY1—chromatin interactions are weakened, suggesting its affinity toward RNA assists in targeting YY1 to chromatin. Knock-down of exosome components results in an increased eRNA half-life and as a consequence a larger spatial reach and a less confined localization of eRNAs to chromatin. Indeed, upregulated and diffuse eRNA localization impairs YY1 binding to its chromatin binding sites. Therefore, RNA and DNA binding by YY1 act cooperatively if co-localized but compete when dispersed (Sigova et al., 2015).

## Co-Transcriptional, RNA-Assisted Formation of Membrane-Less Structures

Similar to the estrogen-induced increase in transcriptional burst size of *Foxc1* upon co-localization with its enhancer and eRNA, genes close to large nuclear speckles containing the non-coding RNA *Malat1* are subject to an increase in burst size and amplify their transcriptional output. After heat shock, *Hsp* transgenes and endogenous genes are induced at the same time irrespective of whether the gene is speckle-associated or not. However, cells with the *Hsp* gene positioned in the vicinity of nuclear speckles surpass cells whose heat-inducible gene is apart from speckles in signal intensity and size of nascent RNA spots. The boosted transcriptional response at speckles correlates with lower exosome activity and larger foci of elongating RNA polymerase II (Khanna et al., 2014; Kim et al., 2020). Furthermore, association of endogenous *Hsp* genes with speckles showed a ripple effect. RNA-FISH of neighboring genes also revealed an increase in the size of transcriptional bursts, but not frequency, when associated with speckles (Kim et al., 2020). Not only are genes more efficiently expressed when in proximity to *Malat1* speckles, but splicing rates are markedly elevated presumably due to an increase in the availability of splicing machinery (Ding and Elowitz, 2019).

Most examples of pc- and eRNAs, mentioned above, likely exert their regulatory roles before decay or diffusion away from their encoding loci. In contrast, in many cell types dozens or more *Malat1*-containing bodies are observed in individual nuclei, which outnumbers the *Malat1* alleles, suggesting these entities are positioned in nuclear space uncoupled from *Malat1*-encoding genes. However, there is evidence of co-transcriptional protein recruitment and body assembly at the gene locus of the architectural RNA. One strategy to assess the potential role of a RNA in co-transcriptional nuclear body formation comprises the artificial tethering of candidate RNAs to an ectopic genome location and monitoring protein recruitment and body formation. Chosen transcripts are tagged with MS2 and co-expressed in cells with a MS2-binding protein fused to LacI. The cells contain a LacO array as the ectopic genome site to concentrate RNA, mimicking endogenous RNA clusters of bursting transcription sites (Shevtsov and Dundr, 2011). Indeed, MS2-tagged *Malat1* efficiently recruits speckle protein markers, such as splicing factor SRSF1, to LacO arrays and forms nuclear puncta (Tripathi et al., 2012).

The following three features of *Malat1* emphasize its potential to assist in membrane-less body formation (Sanford et al., 2009; Tripathi et al., 2010; Larsson et al., 2019): (i) A *Malat1* RNA molecule contains around 50 potential SRSF1-binding sites to concentrate the splicing factor critical for speckle integrity. (ii) The locations of these sites are biased toward the 5’ end of the *Malat1* molecule, the RNA part transcribed first and therefore with the most long-lived chromatin association. (iii) *Malat1* transcription is characterized by one of the largest transcriptional burst sizes (Figure 1D; Larsson et al., 2019). The temporally confined, quasi-synchronous emergence of RNA molecules, as opposed to a steady production of few transcripts at any given time point, likely amplifies the function of *Malat1* to act as a sponge during transcription.

Similarly to *Malat1*, non-coding RNA *Neat1* triggers paraspeckle formation co-transcriptionally (Mao et al., 2011; Shevtsov and Dundr, 2011). Increased RNA abundance at a gene locus as means to initiate sequestration of and multivalent interactions between proteins to form membrane-less structures is reminiscent of ribosomal RNA transcription in nucleolus formation (Hernandez-Verdun, 2011).

## Discussion

Parameters of single-cell and locus-specific transcription, transcript length, RNA decay and diffusion rates all influence the time of chromatin association for nascent, regulatory transcripts and are possibly of equal importance to the structural and motif content of the RNA in order to regulate proximal genes. We speculate that a large burst size combined with increased protein-binding motif occurrence toward the 5’ ends of RNAs is one solution in the parameter space to locally concentrate protein factors with RNA affinity for subsequent, site-specific regulation of nuclear processes. However, the act of transcription itself, independent of an increased abundance of specific RNA motifs, can affect the expression of proximal transcription units to some extent. Indication of regulatory RNA function with little sequence requirements is suggested by the correlation in expression of neighboring genes (Ebisuya et al., 2008). Furthermore, across rodents, expression levels of protein-coding transcripts co-evolve with expression of neighboring non-coding RNAs. Protein-coding RNAs show a narrower distribution of expression levels from different rodents than non-coding RNAs. Therefore, if expression of a non-coding RNA is gained or lost during evolution, the transcriptional output of neighboring protein-coding genes is accordingly found augmented or pruned (Kutter et al., 2012). Despite correlation in the expression levels of non-coding and coding RNAs, nucleotide substitution rates for non-coding transcripts are much faster in comparison to neighboring protein-coding genes, suggesting the contribution of general, in addition to RNA- and motif-specific, protein factors to the regulatory interplay between proximal transcription sites (Ponjavic et al., 2009; Orom et al., 2010). A mechanistic explanation is perhaps provided by interactions between low-complexity C-terminal domains (CTD) of multiple RNA polymerase II complexes and between the CTD and the transcription preinitiation complex (PIC), both interactions increase transcription efficiency and might take place between different but proximal genes or genes and transcribed super enhancers (Quintero-Cadena et al., 2020).

In a model in which co-transcriptional chromatin decoration with RNA overcomes barriers for transcription initiation or repression of proximal genes, protein-coding transcripts cannot be categorically excluded from regulatory roles commonly assigned to non-coding RNAs. Results from studies applying enhancer screening, followed by CRISPR-Cas9 manipulation, or analysis of gene expression levels associated with sequence variation in regulatory regions revealed protein-coding gene promoters as potent distal regulatory elements (Dao et al., 2017; Mitchelmore et al., 2020). The dual role of promoters in the regulation of immediate downstream and distal gene expression is consistent with the notion that protein-coding RNAs, immediately downstream of promoters, might as well be involved in the regulatory process of other, proximal genes. Furthermore, protein-coding RNAs can seed larger, membrane-less structures. Using the MS2-tethering approach histone *H2b* RNA was found capable to induce subnuclear structures resembling histone locus bodies and RNA from a β-globin minigene to assemble splicing speckle components into nuclear puncta (Shevtsov and Dundr, 2011). Chromosome conformation techniques and proximity mapping of pairwise or multiple RNAs simultaneously (Lieberman-Aiden et al., 2009; Morf et al., 2019; Cai et al., 2020) in combination with measurements of transcription output and kinetics might be one way to comprehensively identify regulatory relationships between transcripts and genes. Furthermore, recent advances that allow monitoring of genome architecture and transcription at the single-cell level (Nagano et al., 2013; Stevens et al., 2017; Larsson et al., 2019) will provide further insights into how the interplay between genome structure, RNA, and characteristics of its synthesis, regulates proximal gene transcription with high spatial specificity. Altogether, these findings and new approaches progressively uncover a principle of genome physiology in which RNAs not only comprise its primary output, but simultaneously contribute to the regulatory input for genome expression.

## Author Contributions

All authors contributed to the article and approved the submitted version.

## Conflict of Interest

The authors declare that the research was conducted in the absence of any commercial or financial relationships that could be construed as a potential conflict of interest.
